# Our New York Letter

**Published:** 1888-03

**Authors:** 


### Correspondence.

### OUR NEW YORK LETTER.

DR. HAMILTON ON "THE DETECTION OF CONCEALED INSANITY BY NITROUS-OXIDE GAS AND OTHER ANÆSTHETICS"—THE PROPHYLACTIC TREATMENT OF DIPHTHERIA—INCENDIARY FIRE IN THE 42D STREET HOSPITAL—A SHOCKING ACCIDENT IN ROOSEVELT HOSPITAL—HEBER NEWTON ON THE FAITH CURE.

*To the Editors Atlanta Medical and Surgical Journal:*

A paper, entitled "The Detection of Concealed Insanity by Nitrous-Oxide Gas and other Anæsthetics," was recently read by Dr. Allen McLane Hamilton before the Section in Neurology of the New York Academy of Medicine. The use of anæsthetics for detecting malingering was, he said, an old practice, but as yet this method had not been used in the diagnosis of mental disease. A very common and early effect of anæsthetics is to cause a state of mind similar to that occurring in hypnotism, with abolition of the controlling faculties. During this stage the anæsthetized subject betrays, by the use of language, the formation of coherent ideas relating to his condition or to the operation, but at the same time he may be perfectly unconscious of his surroundings. Dr Hamilton has studied the effects of nitrous-oxide gas for more than ten years, and its value as a diagnostic agent was suggested by a case of insanity with concealed delusions. His experience has been, that where patients tried to conceal mental weakness from fear of being confined in an asylum, the first stage of intoxication would often cause a prompt expression of their delusions or hallucinations. In one patient under his care, who was very nervous and emotional, and also fearful of becoming insane, the most careful examination during repeated conversations failed to detect any intellectual weakness. An attempt was made by the doctor to ascertain if this patient had ever tried to kill herself, but she always denied making any attempt on her life. While under the influence of a mixture of two parts nitrous-oxide and one part air, this patient began talking to herself, saying there was blood on her clothes, and that somebody was going to kill her. When asked whom she referred to, she named her husband and brother. She also admitted having tried to cut a blood-vessel in her arm, and raising her sleeve showed a red scar. In another case of a young girl who had for sometime acted strangely, the doctor was unable to reach any positive conclusion, as during the examination she was always on her guard. He administered the gas to her, and from her conversation which followed, a diagnosis of religious melancholia was made. Each of these cases proved, by their subsequent history, the correctness of Dr. Hamilton's conclusions. As a medico-legal test he considered this plan much superior to hypnotism, which he referred to as unreliable and a scientific amusement. He recommended primary anæsthesia with nitrous-oxide in suspected insanity, and said that much harm might be avoided by an early detection of suicidal and homicidal delusions in people now unconfined.

Dr. A. Caillé recently read a paper before the Academy of Medicine on "A Method of Prophylaxis in Diphtheria." There were, he said, children who were subject to attacks of diphtheria yearly, and his theory was that the diphtheritic germs are retained by these persons in their unhealthy oral or nasal cavities. He had selected eight children for purposes of experiment, who had each had diphtheria two or more times. All with decayed teeth received the proper treatment, and each child used an antiseptic mouth-wash three times daily, besides snuffing some of the fluid into the nose. During the two years that this treatment was continued, none of the children contracted true diphtheria, although several had attacks of tonsilitis and pharyngitis. Prophylactic treatment, he believed, would be of more value to the public than plans for combating the disease after it had already developed. Hypertrophied tonsils, he said, should be reduced, antiseptic gargles used, carious teeth attended to, and children suffering from ordinary sore throats should remain away from school. In discussing this subject, Dr. A. Jacobi, the President of the Academy, said he believed that the diphtheritic germs could remain in the lymphatic glands and mucous membranes, but renewed attacks of the disease might also arise by infection from the furniture of the sick-room. Dr. Joseph E. Winters recommended that where diphtheria existed in a family the well children should take calomel, quinine and large doses of the tinct. ferri chlor.

A few days ago a fire occurred in the Hospital for the Ruptured and Crippled at 42d Street and Lexington avenue. It originated in one of the doctor's rooms, but was soon extinguished. About ten minutes later another fire broke out in the northwest wing of the building, which threatened to result disastrously, but the patients were all removed to neighboring houses in safety and only one person, a servant, lost her life. Several days elapsed, and the inmates had returned to their wards, when the institution was again discovered to be on fire, but the flames were quickly smothered. An investigation revealed the fact that all the mischief was caused by a little girl, aged eleven, an inmate of the hospital, who confessed having set the place on fire each time, but said she didn't know why she did it.

A very unfortunate accident lately occurred at the Roosevelt Hospital, owing to carelessness on the part of an employee. A young man had just undergone a surgical operation, and while still under the influence of ether he was placed on a roller stretcher and removed by an orderly from the operating room to the hall. The door leading to the elevator was then opened by another attendant, and the orderly, supposing the elevator was on that floor, pushed the stretcher into the shaft. The patient fell about twenty-three feet, fracturing his skull and died in about an hour.

The Rev. Heber Newton recently delivered a discourse upon the influence of mind upon physical diseases. Every physician, he said, understood the importance of mental states in bringing about cures, and the patient who does not expect to get well destroys the powers of the best drugs. All diseases, he believes, are now complicated by the excessive nervous development of the present age, and it must be considered sound philosophy that bids us "Seek the highest tonic, not so much in hypophosphites as in joy; the softest opiates, not in bromides, but in the peace that passeth understanding." In his advice to physicians he says: "Our medical faculty, if wise, will not attempt to sneer down a curative force which they have always theoretically admitted, but which they have practically neglected. Rather will they correct the defective methods of a too purely physical science by the supplemental methods of mental science. Let them absorb what is good in this heresy, and thus they will best refute it. Let them enter in their pharmacopœia the tonics of thought and feeling. Let them administer mental and moral stimulants scientifically as those who really believe that an idea may be the most potent of drugs." He then advises the mind-curers to go to school to philosophy and learn to use metaphysics more sanely, and to remember the limitations even of mind when bodied in so very material an organization. They are told not to endeavor to cure everything by one specific, and also to beware of the quacks who gather like the camp-followers of this new movement, looking only for the booty of success. A. F.

February 8, 1888.

